# Astrocytic Expression of GSTA4 Is Associated to Dopaminergic Neuroprotection in a Rat 6-OHDA Model of Parkinson’s Disease

**DOI:** 10.3390/brainsci7070073

**Published:** 2017-06-26

**Authors:** Michael Jewett, Itzia Jimenez-Ferrer, Maria Swanberg

**Affiliations:** Translational Neurogenetics Unit, Wallenberg Neuroscience Center, Department of Experimental Medical Science, Lund University, BMC A10, Sölvegatan 17, 221 84 Lund, Sweden; michael.jewett@med.lu.se (M.J.); itzia.jimenez@med.lu.se (I.J.-F.)

**Keywords:** Parkinson’s disease, Vra1, 6-OHDA, neuroprotection, dopaminergic neurons, GSTA4

## Abstract

Idiopathic Parkinson’s disease (PD) is a complex disease caused by multiple, mainly unknown, genetic and environmental factors. The Ventral root avulsion 1 (*Vra1*) locus on rat chromosome 8 includes the Glutathione S-transferase alpha 4 (*Gsta4*) gene and has been identified in crosses between Dark Agouti (DA) and Piebald Virol Glaxo (PVG) rat strains as being associated to neurodegeneration after nerve and brain injury. The *Gsta4* protein clears lipid peroxidation by-products, a process suggested to being implicated in PD. We therefore investigated whether PVG alleles in *Vra1* are neuroprotective in a toxin-induced model of PD and if this effect is coupled to *Gsta4*. We performed unilateral 6-hydroxydopamine (6-OHDA) partial lesions in the striatum and compared the extent of neurodegeration in parental (DA) and congenic (DA.VRA1) rats. At 8 weeks after 6-OHDA lesion, DA.VRA1 rats displayed a higher density of remaining dopaminergic fibers in the dorsolateral striatum compared to DA rats (44% vs. 23%, *p* < 0.01), indicating that *Vra1* alleles derived from the PVG strain protect dopaminergic neurons from 6-OHDA toxicity. *Gsta4* gene expression levels in the striatum and midbrain were higher in DA.VRA1 congenic rats compared to DA at 2 days post-lesion (*p* < 0.05). The GSTA4 protein co-localized with astrocytic marker GFAP, but not with neuronal marker NeuN or microglial marker IBA1, suggesting astrocyte-specific expression. This is the first report on *Vra1* protective effects on dopaminergic neurodegeneration and encourages further studies on *Gsta4* in relation to PD susceptibility.

## 1. Introduction

Parkinson’s disease (PD) is a progressive neurodegenerative disease characterized by the loss of dopaminergic neurons in the substantia nigra pars compacta (SNpc) and a range of motor and non-motor symptoms. Around 10% of PD cases are monogenic with a familial inheritance of specific mutations in genes such as α-synuclein (SNCA) and Leucine-rich repeat kinase 2 (LRRK2) [1]. The remaining 90% of PD cases are idiopathic, where the etiology is complex with a combination of several genetic and environmental risk factors. In addition, gene-gene and gene-environment interactions influence the risk for idiopathic PD, e.g., by affecting susceptibility to toxins [2,3]. Unidentified genetic risk factors were recently estimated to account for 40% of the variation in PD risk [4]. Despite the identification of 24 risk loci through meta-analysis of genome-wide association studies (GWAS) [5], there is still a large missing heritability, i.e., unknown genetic risk factors, in PD [6].

One tool to identify genetic factors behind complex diseases is linkage analysis, an unbiased method that links specific genetic regions to naturally occurring phenotypic variance across inbred rodent strains [7]. Once these genetic regions are identified, functional experimental analyses can be used to examine the impact of these regions on disease processes. The *Vra1* region was originally identified by linkage analysis in a cross between inbred Dark Agouti (DA) and Piebald Virol Glaxo (PVG.1AV1) rats as linked to neurodegeneration after ventral root avulsion (VRA) [8]. Rats carrying PVG.1AV1 alleles in the *Vra1* region were back-crossed multiple times to DA to create the DA.VRA1 congenic strain, carrying PVG.1AV1 alleles in the neuroprotective *Vra1* region on a DA strain background. This strain was used to finely map *Vra1*, and several candidate genes were discovered [9]. A later study determined Glutathione *S*-transferase alpha 4 (*Gsta4*) as the strongest candidate gene regulating neurodegeneration in response to VRA [10] and traumatic brain injury (TBI) in DA.VRA1 congenic rats [11].

GSTA4 is part of a large family of glutathione *S*-transferase (GST) isoenzymes, which are important for cellular detoxification [12]. GSTs are divided in distantly related subgroups (class alpha, mu, pi, sigma, and theta), and alpha class GSTs are among the most abundant in mammals [13]. GSTA4, an alpha class GST, is involved in oxidative stress by clearing toxic lipid peroxidation by-products such as 4-hydroxynonenal (HNE) through their conjugation to glutathione (GSH) [10]. Oxidative stress mechanisms, including lipid peroxidation, are heavily implicated in several neurodegenerative diseases, including PD, making GSTA4 an interesting target in relation to PD. In support of this, HNE has been shown to be significantly elevated in PD brains, while GSH is reduced in the same context [14,15,16]. Since GSTs are critical for clearance of HNE and maintenance of GSH, their activity could be essential in slowing down disease progression.

6-hydroxydopamine (6-OHDA) a hydroxylated analogue of dopamine that is readily taken up by dopaminergic cells and oxidized in the cytosol leading to oxidative stress mechanisms [17], is widely used as a neurotoxin in PD research [18,19,20]. Intraparenchymal injections of 6-OHDA to the brain specifically target the nigrostriatal pathway, making 6-OHDA a viable tool for studying neurodegenerative processes, especially those related to PD and oxidative stress. Moreover, 6-OHDA has been shown to increase the levels of HNE in the striatum of rodents, peaking 1 day after injury and returning to baseline at 7 days post injury, suggesting a potentially protective role for GSTA4 in the 6-OHDA PD model [21]. We therefore hypothesized that the *Vra1* locus encoding *Gsta4* in the DA.VRA1 strain protects the nigrostriatal pathway after unilateral striatal 6-OHDA injections. To test this hypothesis, we compared the extent of dopaminergic neurodegeneration in the striatum and SNpc in DA and DA.VRA1 rats. We also analyzed *Gsta4* gene expression levels and determined cell-type specific GSTA4 protein expression. Our results show that PVG.1AV1 alleles in *Vra1* are neuroprotective in the 6-OHDA PD model, and suggest that this effect is mediated by increased GSTA4 expression in astrocytes.

## 2. Materials and Methods

### 2.1. Experimental Design

**Figure 1:**
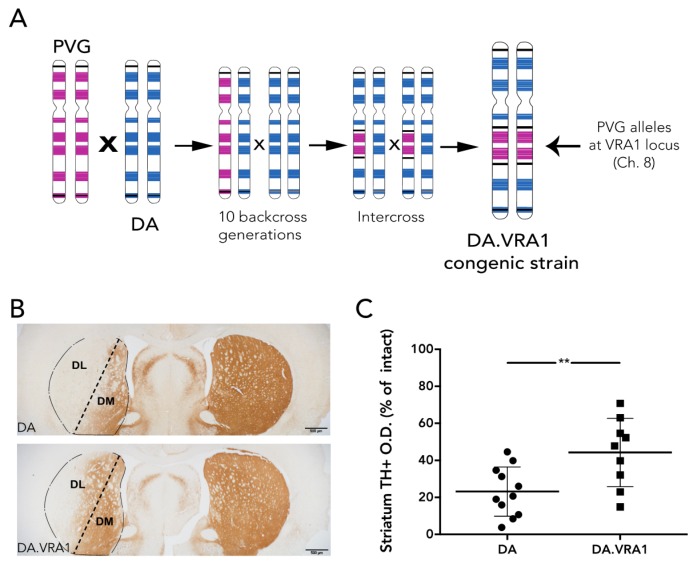
The *Vra1* locus confers protection of striatal dopaminergic fibers after 6-OHDA lesion. (**A**) Breeding schematic for the DA.VRA1 congenic strain: PVG.1AV1 was back-crossed to DA 10 times with selection on the *Vra1* region; an intercross generated the homozygous DA.VRA1 congenic strain, with >99.9% DA genome outside of the congenic fragment. (**B**) Representative pictures showing coronal sections of the striatum from each strain stained for Tyrosine Hydroxylase (TH). Scale bar = 500 μm The lesioned striatum is divided in two parts: DL, the region receiving most afferent projections from the cells of the substantia nigra pars compacta, and DM. (**C**) Optical density (O.D.) quantification of TH+ fibers in the lesioned DL relative to the intact DL striatum at 8 weeks post-surgery. DA.VRA1 rats display a higher density of TH+ fibers in the lesioned relative to intact DL striatum, suggesting increased dopaminergic fiber survival compared to DA. Mean ± SD, ** *p* < 0.01 after an unpaired *t*-test. (DA = Dark Agouti; DA.VRA1 = *Vra1* congenic; DL = Dorsolateral; DM = Dorsomedial)

All animals were housed 2–3 per cage and given *ad libitum* access to food and water during a 12 h light/dark cycle. A cohort of 56 female rats was used in this study (28 DA and 28 DA.VRA1 congenics). Founders for each strain, both of which are inbred, were bred and kindly provided by Professor Pielh at the Karolinska Institutet, Stockholm, Sweden. The DA.VRA1 congenic rats were originally bred as previously described [10]: male rats with PVG alleles in the *Vra1* locus on chromosome (chr) 8 were repeatedly backcrossed to the DA strain in order shorten the *Vra1* fragment and reduce the number of DA alleles outside *Vra1* (RNO8; D8Rat24-D8Got132; 82.2–88.6 Mb) to <0.1% of the resulting DA.VRA1 congenic strain genome (Figure 1A). The rats were subjected to 6-OHDA lesion at 13 weeks of age, corresponding to 200–220 g body weight. 12 DA, and 12 DA.VRA1 rats were sacrificed at 8 weeks post-surgery for histological analysis, while 16 DA and 16 DA.VRA1 rats were sacrificed at 2 or 7 days post-surgery for gene expression analysis. All procedures described were approved by the Ethical Committee for the use of laboratory animals in the Lund/Malmö region.

### 2.2. Surgical Procedure

Rats were anaesthetized with isofluorane (Apoteksbolaget, Stockholm, Sweden) and placed in a stereotaxic frame with a flat-skull position. The top of the head was shaved, followed by a 0.2 mL s.c. injection of Marcain (Apoteksbolaget, Stockholm, Sweden). After making a fine 5 mm cut down the midline of the scalp, three small holes were drilled in the skull reaching the dura mater. Unilateral injections of 6-OHDA (Sigma-Aldrich, Gillingham, UK) (3.5 mg/mL dissolved in a solution of 0.9% saline with 0.02% ascorbic acid) were made in the dorsal striatum using a 10 µL Hamilton syringe fitted with a glass capillary (outer diameter of 250 µm). Three injections of 2 µL each adding up to 21 µg were performed at the following coordinates, given in mm relative to bregma and dural surface [22]: (i) AP = +1.0, ML = −3.0, DV = −5.0; (ii) AP = −0.1, ML = −3.7; DV = −5.0; (iii) AP = −1.2, ML = −4.5, DV = −5.0. Surgical clips were used for closing the incisions. After the procedure, 0.15 mL Metacam (Apoteksbolaget, Stockholm, Sweden) was injected s.c. for post-operative analgesia. All animals were then placed in clean cages on a heated pad for recovery.

### 2.3. Tissue Preparation and Histology

Animals for histological analysis were sacrificed 8 weeks post-surgery. Rats were sedated by i.p. injection of 0.7 mL sodium pentobarbital (Apoteksbolaget, Stockholm, Sweden), before being perfused through the ascending aorta with 100 mL saline (0.9% NaCl) at room temperature, followed by 250 mL ice-cold paraformaldehyde (4% in pH 7.4 phosphate-buffered saline (PBS)). Brains were removed and post-fixed in 4% paraformaldehyde (PFA) overnight, cryoprotected in 30% sucrose (in PBS, with 0.01% sodium azide), then sectioned coronally on a freezing microtome (Microm HM 450, Thermo Scientific, Waltham, MA, USA) at 40 µm. Immunohistochemical stainings were performed on free-floating sections at room temperature using a mouse anti-tyrosine hydroxylase (TH) primary antibody (1:1000 Immunostar, Hudson, WI, USA). The SNpc sections were given an initial antigen-retrieval incubation in Tris/EDTA (pH 9.0) at 80 °C for 45 min, washed in PBS before and after. All sections were quenched with 3% H_2_O_2_/10% MetOH for 30 min, and then placed in a blocking solution with 5% Normal Horse Serum (NHS) and 0.3% Triton-X-100 in PBS (PBS-T) for 1 h before overnight primary antibody incubation. Following three washes in PBS-T, sections were incubated with biotinylated secondary antibody (horse anti-mouse 1:200, Vector Laboratories, Burlingame, CA, USA) for 1 h. This was followed by three PBS-T washes and a 30 min incubation with an avidin-biotin peroxidase solution (ABC Elite, Vector Laboratories, Burlingame, CA, USA). After three PBS washes, the antigen was visualized using 3,3′-diaminobenzidine-tetrahydrochloride-dihydrate (DabSafe, Saveen Werner, Sweden) as a chromogen and washed three times in PBS. Stained sections were mounted on glass slides, dehydrated with increasing concentrations of ethanol and pure xylene, and finally coverslipped using DPX mounting medium (Sigma-Aldrich, Gillingham, UK).

Double immunofluorescence stainings were performed as described above without the antigen retrieval and quenching steps. Blocking and primary antibody incubations were performed with 10% normal donkey serum (NDS) in PBS-T. The primary antibodies used were rabbit anti-GSTA4 (1:100 Antibodies-online GmbH, Aachen, Germany), mouse anti-GFAP (1:1000 Santa Cruz, Santa Cruz, CA, USA), and anti-IBA1 (1:500 Synaptic Systems, Göttingen, Germany), (NeuN 1:1000 Millipore, Billerica, MA, USA) and were incubated together at 4 °C, then Cy-3 and Alexa 488-conjugated secondary antibodies (1:500 Jackson Immunoresearch, Suffolk, UK) were incubated simultaneously for 1.5 h at room temperature. A short 10-min incubation in DAPI (1:1000 Sigma-Aldrich, Gillingham, UK) was added as a last step. After mounting the sections on glass slides and waiting 15–20 min for the sections to dry, the slides were coverslipped with PVA-DABCO (Sigma-Aldrich, Gillingham, UK).

### 2.4. Quantification of Dopaminergic Fiber Loss in the Striatum

Striatal TH+ fiber optical density (O.D.) was measured by image densitometry at three coronal levels (+1.60, +0.70, −0.30 mm from bregma) of the dorsolateral (DL) striatum (striatum division between DL and dorsomedial (DM) is shown in Figure 1B) using the ImageJ software (https://imagej.nih.gov NIH, USA). Each image was analyzed using the O.D. values obtained from the Rodbard calibration curve after being transformed into 8-bit images (gray-scale). The software then calculated the mean gray value based on the strength of the TH+ staining. The corpus callosum O.D. values were used to correct for non-specific background staining. Pictures of the striatum were taken at 2× magnification using a bright field microscope (Olympus BX53, Tokyo, Japan) linked to a high-resolution camera (Olympus DP73, Tokyo, Japan) and cellSens Dimension software (Olympus, Tokyo, Japan). The data is presented relative to the corresponding area from the intact side. Of 24 animals, 4 were excluded from the analysis due to complications during surgery or with tissue processing, leaving 11 DA, and 9 DA.VRA1 for quantification.

### 2.5. Quantification of Dopaminergic Cell Loss in SNpc

Dopaminergic neurons in the SNpc were quantified by stereology of TH+ cells according to the optical fractionator principle as described previously [23]. Every fourth section (section sampling fraction, *ssf* = 4) covering the full extent of the SNpc (−4.04 to −7.56 from bregma) was sampled for analysis, yielding 10–12 sections per animal. The average mounted section thickness (h) was 24.1 μm (±2.3) and no guard zones were used (thickness sampling fraction (tsf) = 1). Section thickness was measured at every fourth site while counting, and the area-sampling fraction (asf) was on average 0.112. Dissector volume (h*A_frame_) was 86,400 mm^3^ on average, and the average number of dopaminergic neurons counted in each individual was 210 (±41). A maximal Gundersen coefficient of error (CE) [24] of 0.08 was accepted. For cells to be counted, we relied on the following criteria: the cell body had to be clearly defined by the TH+ marker with a visible lighter-stained nucleus; if cells were too dark and the nucleus was not clearly visible, they were still counted if the projections were distinctly visible as being part of their respective cell bodies. Of 24 animals, 6 were excluded from the analysis due to complications during surgery or with tissue processing, leaving 10 DA, and 8 DA.VRA1 for quantification. When correlating stereological cell counts with fiber O.D., 10 DA and 7 DA.VRA1 animals were included.

### 2.6. Gene Expression Analysis

Animals were sedated and sacrificed as described above at 2 days or 7 days post-surgery. Only 100 mL ice-cold saline was used for the perfusion step. Brains were quickly extracted and placed with the ventral side up in a rat brain slice matrix on ice. Five coronal cuts separating the forebrain and the midbrain from other brain areas were made with fine razor blades (3 cuts to extract the striatum, 2 for the midbrain). Corresponding brain slices were placed on a glass plate on ice where 20–30 mg pieces of right and left striatum and ventral midbrain were dissected from the surrounding tissue and placed in separate lysing matrix beaded tubes (MP Biomedicals, Burlingame, CA, USA). The tubes were transferred to dry ice and subsequently stored at −80 °C. RNA isolation was performed using the RNeasy Mini kit (Qiagen, Hilden, Germany), following the supplier’s protocol from steps 4 to 7. The first three steps were substituted by adding 600 µL Trizol (Life Technologies, Warrington, UK) to each sample before a 20 s homogenization step with a FastPrep homogenizer (MP Biomedicals, Burlingame, CA, USA). The samples were then transferred to a clean Eppendorf tube and resuspended in 0.2 mL chloroform/mL Trizol, shaking vigorously, and centrifuged for 15 min at 12,000 *g* (4 °C). RNA concentration was determined using NanoDrop (Thermo Scientific, Waltham, MA, USA). Reverse Transcription was performed using the SuperScript^®^ III First-Strand Synthesis System (Invitrogen, Waltham, MA, USA) according to the manufacturer’s protocol. Quantitative Polymerase Chain Reaction (qPCR) was conducted using the SSo Advanced Universal SYBR green Supermix (BioRad, Hercules, CA, USA) according to this protocol: 5 μL Supermix + 1 μL nuclease-free H_2_O + 0.5 μL of each primer + 3 μL cDNA for each sample. Amplification was performed with a 40 cycle, 3-step PCR protocol (1. 30 s at 95 °C; 2. 60 s at 64 °C; 3. 5 min at 68 °C) with the following primers: *Gsta4* (fw: GACCGTCCTGAAGTTCTAGTGA ,rev: TGCCTCTGGAATGCTCTGT), *gapdh* (fw: CAACTCCCTCAAGATTGTCAGCAA, rev: GGCATGGACTGTGGTCATGA), and *β*-*actin* (fw: AAGTCCCTCACCCTCCCAAAAG, rev: AAGCAATGCTGTCACCTTCCC).

### 2.7. Statistical Analysis

Statistical tests were performed using the GraphPad Prism software (version 6, La Jolla, CA, USA). Stereology and densitometry differences between groups were analyzed using unpaired *t*-test; statistical significance was set at *p*-value < 0.05 and values are expressed as mean ± standard deviation (SD). Correlation analysis was performed using the Pearson correlation coefficient (*r*), statistical significance was set at *p*-value < 0.05, and a 95% confidence interval was used. A one-way analysis of variance (ANOVA) test was used to calculate gene expression differences between groups at each time point. A two-way ANOVA was used to calculate gene expression differences between two time-points within groups. Both ANOVAs were followed by post-hoc Bonferroni’s multiple comparisons test.

## 3. Results

### 3.1. 6-OHDA Induces Less Dopaminergic Fiber Loss in DA.VRA1 Congenic Rats

We used the three-site unilateral striatal 6-OHDA model as previously described by Kirik et al. [25]. This model was chosen since it has been shown to distribute the toxin throughout the striatum and produce partial and progressive degeneration of dopaminergic fibers in the striatum and cell bodies in the SNpc at 8 weeks after injections. Staining of the TH+ fibers in the striatum showed that mainly the DL area was affected. The striatum was therefore subdivided into DL, the area mainly innervated by the neurons of the SNpc sensory-motor innervation, and dorsomedial (DM), which is mainly innervated by the ventral tegmental area (VTA) [26,27] (Figure 1B). Optical densitometry measuring the remaining TH+ fibers of the ipsilateral (IL) compared to the contralateral (CL) striatum showed a higher proportion of remaining TH+ fiber density in the IL DL striatum of DA.VRA1 compared to DA rats. ({mean (SD)} 44 (18)% vs. 23 (13)%, *p* < 0.01, Figure 1C). PVG.1AV1 alleles in the *Vra1* locus thus protected DA.VRA1 congenic rats from 6-OHDA induced loss of striatal dopaminergic fibers.

### 3.2. Striatal Dopaminergic Fiber Loss Correlates to Cell Loss in SNpc

**Figure 2:**
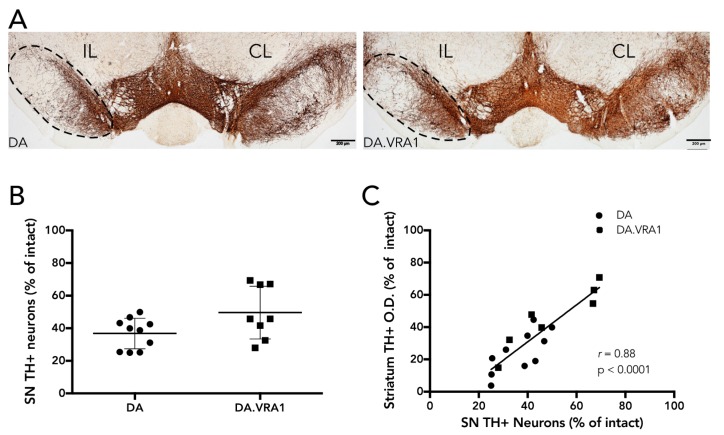
Loss of dopaminergic cells in the SNpc 8 weeks after striatal 6-OHDA lesion. (**A**) Representative images showing the loss of TH+ cells in SNpc of DA and DA.VRA1 rats. Delineated areas outline the SN IL to the lesion. Scale bar = 500 μm. (**B**) Stereological quantification of TH+ neurons in the SNpc at 8 weeks post 6-OHDA lesion. Individual data points and mean ± SD are shown after unpaired *t*-test (*p* = 0.052). (**C**) The Pearson correlation coefficient (*r*) shows that the percentage of remaining TH+ cells in the SNpc strongly correlates with the density of TH+ fibers in the DL striatum on the IL side. (DA = Dark Agouti; DA.VRA1 = *Vra1* congenic; CL = Controlateral; IL = Ipsilateral)

Stereological cell counts of TH+ neurons of the SNpc 8 weeks post 6-OHDA lesion showed a reduction in dopaminergic cells in the SNpc IL to the striatal 6-OHDA lesion in both strains (Figure 2A,B). There was no significant difference in cell loss between the strains, but a trend reflecting the densitometry results was observed with DA.VRA1 animals displaying a milder lesion compared to DA (49 (16)% vs. 39 (9)% TH+ cell survival, *p* = 0.052, Figure 2B). In individual rats, there was a strong positive correlation between IL DL striatal TH+ fiber density and remaining dopaminergic cells in the SNpc (*p* < 0.0001; *r* = 0.88, Figure 2C). Loss of dopaminergic fibers in the striatum thus reflects the extent of dopaminergic neurodegeneration in both the DA and DA.VRA1 strain.

### 3.3. Gsta4 Expression is Increased in DA.VRA1 Rats 2 Days after 6-OHDA Lesion

**Figure 3:**
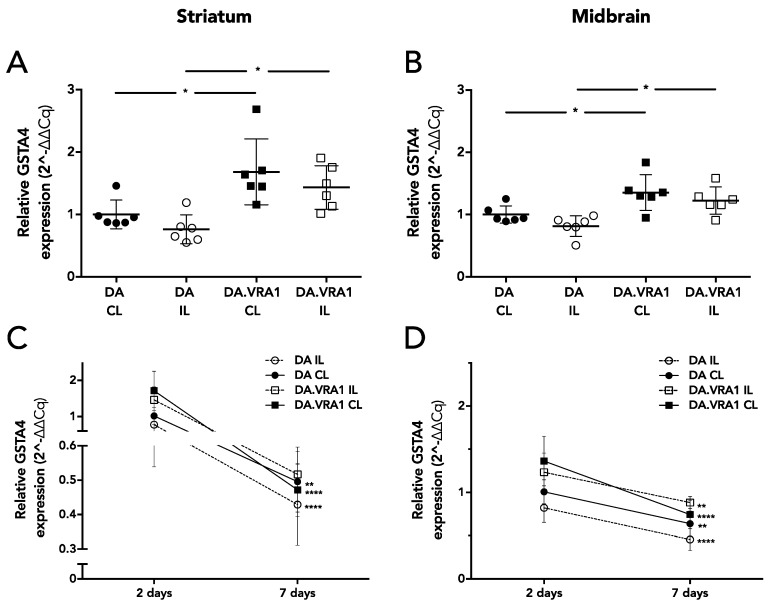
*Gsta4* gene expression in the striatum and midbrain after striatal 6-OHDA lesion. At 2 days post lesion, *Gsta4* expression is significantly higher in DA.VRA1 compared to DA for both the ipsilateral and the contralateral sides of the striatum (**A**) and midbrain (**B**). *Gsta4* expression levels in the striatum (**C**) and midbrain (**D**) are higher at 2 days compared to 7 days post lesion in both strains. *p* values relate to the comparisons between 2 and 7 days for each specific strain and side. Gene expression levels are related to the mean value for DA at the IL side. * *p* < 0.05 for (**A**) and (**B**) based on a one-way ANOVA, mean ± SD are shown. ** *p* < 0.01, **** *p* < 0.0001 for (**C**) and (**D**) based on a two-way ANOVA. (DA = Dark Agouti; DA.VRA1 = *Vra1* congenic; CL = Controlateral; IL = Ipsilateral)

*Gsta4* is a transcript that has been reported as being strongly *cis*-regulated i.e., encoded and transcriptionally regulated by *Vra1* [10]. *Gsta4* has been identified as the gene in the *Vra1* locus regulating the susceptibility to VRA and TBI [10,11]. We assessed the gene expression of *Gsta4* in the midbrain and striatum at 2 and 7 days after striatal 6-OHDA lesion. The two time points were chosen based on previous studies showing that a glial response to 6-OHDA is evident 2 days after lesion, while significant dopaminergic fiber loss is evident from 6 to 7 days after lesion [18,28,29]. The selected time points thus reflect a sub-acute and an early phase of the degenerative process in the SNpc. At 2 days, we found a significantly higher expression of *Gsta4* in both the IL and CL sides of the striatum (Figure 3B) and midbrain (Figure 3B) of DA.VRA1 compared to DA rats (*p* < 0.05). There were no differences in *Gsta4* expression between the strains at 7 days (Figure S1a,b). *Gsta4* expression levels were higher at 2 days in both strains, and decreased at 7 days in the striatum (DA CL *p* < 0.01, DA.VRA1 IL and CL *p* < 0.0001, Figure 3C) and midbrain (DA IL and CL *p* < 0.01, DA.VRA1 IL *p* < 0.01; CL *p* < 0.0001, Figure 3D).

### 3.4. GSTA4 is Expressed by Astrocytes after 6-OHDA Lesion

**Figure 4:**
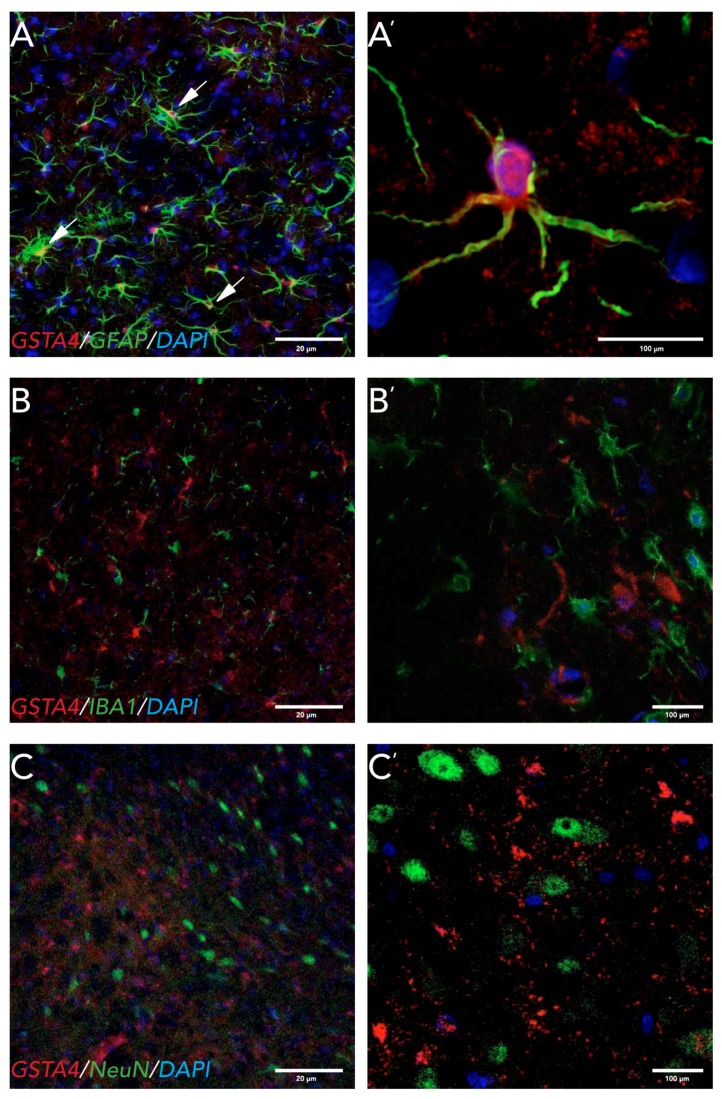
Expression of GSTA4 in midbrain astrocytes 8 weeks after striatal 6-OHDA lesion. Immunofluorescent staining of GSTA4 combined with cell-specific markers for (**A**) astrocytes; GFAP, (**B**) microglia; IBA1 and (**C**) neurons; NeuN. GSTA4 staining co-localized with GFAP (**A’**) but not IBA1 (**B’**) or NeuN (**C’**), suggesting astrocytic expression. (**A**–**C**) Pictures taken at 20×; scale bar = 20 μm. (**A’**) Zoomed in 60× image showing co-localization, with GSTA4 mainly expressed in the soma. Scale bar = 100 μm. (**B’**, **C’**) Pictures taken at 60×, scale bar = 100 μm. All markers were combined with nuclear marker DAPI (blue).

To assess which cell type in the midbrain expresses GSTA4 protein after the 6-OHDA lesion, we performed double fluorescence immunostaining with astrocytic (GFAP), microglial (IBA1), and neuronal (NeuN) markers 8 weeks after 6-OHDA lesion (Figure 4).

In the IL midbrain, GSTA4 co-localized with GFAP (Figure 4A’) but not IBA1 (Figure 4B’) or NeuN (Figure 4C’), reflecting astrocytic expression. The GSTA4 staining was predominantly seen in the astrocytic cell soma (Figure 4A’). Similar results were seen for the CL midbrain and in the striatum (Figure S2).

## 4. Discussion

Idiopathic PD is a complex disease, which means that both environmental and genetic factors determine disease susceptibility. Environmental factors, such as exposure to pesticides or other toxins, have been clearly identified as contributors to increasing the risk of developing PD [30,31]. Moreover, differential responses to environmental factors are influenced by the interactions of multiple genes, making risk factors more challenging to pinpoint [32]. Identification of genetic risk factors for complex disorders like idiopathic PD can be hypothesis-driven, such as association studies of candidate genes in clinical case-control materials or studies of transgenic animals. However, in order to perform an unbiased search for disease-associated genes, GWAS or whole-genome linkage analyses are required. In GWAS, millions of single-nucleotide polymorphisms (SNPs) are analyzed in large groups of patients and controls. By meta-analysis of GWAS in PD, 24 loci including *SNCA* and *LRRK2,* which are also involved in monogenic forms of PD, have been identified as genetic risk factors for idiopathic PD [5].

Linkage analysis is based on the co-segregation of specific genetic markers with a disease phenotype, and identifies quantitative trait loci (QTLs) for a specific phenotype [7]. Linkage analysis in mouse PD models have identified several QTLs linked to midbrain neurodegeneration [23,33,34]: we recently performed linkage analysis in an F2 intercross between C57Bl/6N and Swiss-OF1-En1+/− mice, identifying multiple QTLs linked to susceptibility of mice to midbrain neurodegeneration and striatal fiber dysfunction [23]. Two previous studies of susceptibility to toxin-induced dopaminergic neurodegeneration performed linkage analysis in N2 generations obtained from intercrossing and subsequent back-crossing of C57Bl/6J to Swiss-Webster mice. The first study identified *Mptp1*, a locus on chr 1 containing 66 known genes including presenilin 2 (*Psen2*) as conferring strain sensitivity to the MPTP neurotoxin [33]. The second identified two QTLs, one on chromosome 5 and one on chromosome 14 as linked to susceptibility to paraquat, a herbicide highly associated with PD risk [34]. One of the genes in the chromosome 5 QTL is the *Gstm1* pseudogene, a member of the GST superfamily.

No QTL thus far has been identified in rat PD models, but susceptibility to neurodegeneration has been studied in rats after the nerve injury VRA [9]. The QTL *Vra1* on rat chromosome 8 was identified as being linked to neuroprotection after VRA [9] and also shown to confer protection to TBI [11]. In *Vra1*, *Gsta4* was found to be the main candidate gene for conferring neuroprotection through a *cis*-effect on gene expression, likely caused by polymorphisms within or near *Gsta4* affecting gene transcription [9,10,11]. GSTA4 regulates lipid peroxidation, and since there is evidence that lipid peroxidation, and oxidative stress in general, are involved in PD [15,35,36,37], we hypothesized that *Vra1* would have a neuroprotective effect in PD. To test this hypothesis, we studied the response to 6-OHDA-induced partial lesion of the rat nigrostriatal pathway in DA and DA.VRA1 congenic rats [38].

The 6-OHDA rat model has been extensively studied to mimic one of the main pathological processes of PD: the loss of dopaminergic neurons and fibers in the nigrostriatal pathway. The degenerative process is thought to be due to the accumulation of reactive oxygen species (ROS) within the cells [18]. This model has two main variants depending on the target of injection of the toxin: medial forebrain bundle (MFB), or caudate-putamen (CPu). The unilateral MFB lesion has been the most targeted in pre-clinical research due to its strong efficacy in almost completely destroying the nigrostriatal pathway. This type of lesion creates phenotypes that mirror very late stages of PD in patients [28]. Both cognitive and motor behaviors can be readily studied thanks to the complete impairment of the IL nigrostriatal pathway, but due to the extent of degeneration, studying neuroprotective agents or alleles becomes close to impossible [25]. To study mild to moderate parkinsonian phenotypes (early stage parkinsonism) and the effect of neuroprotective agents or alleles, lesion of the dorsal striatum, which leads to a progressive degeneration of the nigrostriatal pathway, is preferable [28]. Kirik et al. have established parameters for a stable unilateral 6-OHDA lesion of the DL striatum with partial degeneration of the nigrostriatal pathway that leaves room for studying the differences between strains at a histological level. The distribution of 21 μg 6-OHDA over three sites in the CPu produced 60–65% dopaminergic fiber loss and about 50% dopaminergic cell loss in the SNpc at 8 weeks post injection in adult Sprague Dawley rats [25]. This is similar to our results from the DA.VRA1 strain (56% fiber loss and 51% cell loss), while we found an increased susceptibility to 6-OHDA lesion in the DA strain (77% fiber loss and 61% cell loss). Whether the DA.VRA1 and Sprague Dawley rat strains both express higher levels of GSTA4 compared to DA, or if other factors underlie the difference between Sprague Dawley and DA rats remain to be answered. It should be pointed out that Kirik et al. found similar levels of dopaminergic degeneration already at 3 weeks post injection with this protocol, but the extent of degeneration was shown to be close to 10% higher at 8 weeks, indicating that loss of nigral neurons is still progressing within that time. Another point in favor of the 8-week time point comes from results obtained by Sauer and Oertel [39]. They found no significant differences in nigral TH immunoreactivity at 8 weeks compared to 4 weeks, but they did see differences at those time points when marking nigral neurons with Fluorogold retrograde tracer, which they argue is more indicative of the accurate nigral cell number. Since a study of this type has never been done on DA rats, and given the results from studies just mentioned, we opted for the longer post-injection period to detect possible neuroprotective effects in the DA.VRA1 rats.

Our results thus show that compared to DA, PVG alleles in the *Vra1* locus protect DA.VRA1 congenic rats from loss of dopaminergic fibers in the striatum after unilateral striatal 6-OHDA injections. The DA.VRA1 strain displayed an almost 2-fold higher striatal dopaminergic fiber density compared to the DA strain at 8 weeks after 6-OHDA lesion. A similar trend of neuroprotection in the DA.VRA1 congenic strain was found for stereological cell counts of TH+ neurons in the SNpc, with a higher proportion of surviving cells in the congenic strain. Although there was a strong correlation between striatal fiber density and dopaminergic cell numbers in the SNpc, there were only significant differences between the two strains in striatal fiber density and not for nigral cell counts. This could be due to GSTA4 affecting an ongoing degenerative process that starts at dopaminergic projections in the striatum, and has a delayed, retrograde effect on cell somas in the SNpc. Studying strain differences in nigral dopaminergic cell counts at additional, and later, time point would be required to confirm this.

To further investigate the neuroprotective effects seen on striatal dopaminergic fibers in the DA.VRA1 congenic strain, we analyzed mRNA expression of *Gsta4,* the candidate gene from the *Vra1* locus shown to confer neuroprotection after VRA and TBI. *Gsta4* plays an important role in GSH metabolism, which is heavily involved in clearing of oxidative stress and lipid peroxidation products [40]. One of the lipid peroxidation by-products, HNE, has been found to be elevated in PD patients [16]. Ström et al. found the highest levels of *Gsta4* expression between 1 and 5 days after nerve injury in DA and PVG.1AV1 rats [10]. To investigate the role of *Gsta4* in the 6-OHDA model, we analyzed gene expression at 2 and 7 days post lesion. Our results show that *Gsta4* expression is higher in both IL and CL striatum and midbrain of DA.VRA1 compared to DA rats at 2 days post 6-OHDA lesion. However, the difference in gene expression of *Gsta4* between DA.VRA1 and DA rats is smaller (1.3–1.5 fold at 2 days) compared to that seen after nerve injury (3 fold at 5 days). We did not observe any strain differences in *Gsta4* expression levels at 7 days after 6-OHDA lesion, suggesting that GSTA4 exerts its neuroprotective effects within the first days after 6-OHDA lesion. This is supported by previous studies, showing high levels of striatal free radical species already at 25 min post lesion, which drop to control levels 7 days after 6-OHDA lesion [38], and increased HNE levels at 1 day post 6-OHDA injection [21].

The *Vra1* locus in the DA.VRA1 congenic strain used here is flanked by the two genomic markers D8Rat26 (79.35 Mb) and D8Rat75 (89.56 Mb). Expression-QTL analysis showed that *Gsta4* expression displayed by far the strongest linkage to both markers [10]. A few additional *cis*-regulated transcripts were identified as mapping to one of the two markers at *p* < 0.01, but are located outside the *Vra1* fragment. This means they have the same alleles in the DA and DA.VRA1 strains and are therefore discarded as possible candidates for the observed neuroprotective phenotype in VRA, TBI and 6-OHDA lesion. The expression-QTL analysis also revealed several transcripts *trans*-regulated by both markers, including urocortin (*Ucn*), dual oxidase 1 (*Duox1*), copine 6 (*Cpne6*), and chemokine ligand 2 (*Ccl2*) [10]. Of these, *Duox1* and *Ccl2* are the most relevant for studying susceptibility to neurodegeneration; *Duox1*, encodes the enzyme Dual oxidase 1, which is involved in the production of ROS, and thus belongs to the same pathway as 4-HNE [10]; *Ccl2* plays a crucial role in the activation of monocytes and microglia, and has been found to be upregulated in an MPTP PD model [41]. Some significant (*p* < 0.01 cutoff) transcripts were found as being *trans*-regulated by only one of the markers: *Kif11*, *Tap2*, *Hoxc11*, RGD1560859 (*Asprv1*), and LOC100360609 (*Olfr1055*). *Kif11* (known as *Eg5* in humans) is a member of the kinesin family involved in mitotic spindle formation; a recent study has shown that *Eg5* expression levels are regulated by Parkin, which is an E3 ubiquitin ligase heavily involved in PD [42]. Therefore, several other genes may be involved in determining the neuroprotective effects conferred by *Vra1* and should thus be taken into consideration for future PD susceptibility studies. The *Gsta4*-null mouse, generated by Engel et al. [43], has been used by several studies to better understand the role of *Gsta4* in oxidative stress mechanisms [43,44,45,46]. In one of these studies in particular, McElhanon et al. [44] found that the *Gsta4*-null mouse embryonic fibroblasts (MEF) are more sensitive to the PD-associated toxin paraquat.

There are some conflicting reports on the cellular localization of GSTA4 in the CNS. A study in Alzheimer’s disease patient tissue found GSTA4 expressed in neurons and in blood vessels [47]. In rats, GSTA4 has been found in motorneurons of the ventral horn of the spinal cord after VRA [10] and in astrocytes in the cerebellum and neocortex [12]. We found GSTA4 expression in astrocytes both in the striatum and in the SNpc, but not in microglia or neurons at 8 weeks post 6-OHDA lesions. Astrocytes play a pivotal role in the clearing of oxidative stress products and therefore neuroprotection [48]. Astrocytes also have a very high activity of GSH, which uses GSTA4 to degrade HNE [49,50]. One mechanism by which astrocytes might be neuroprotective is through the release of GSH by astrocytes to neurons, which has been seen after nitric oxide (NO) exposure to astrocytes in vitro [50]. A question remaining to be answered is whether the strain differences in *Gsta4* expression observed here affect the metabolism of the 6-OHDA toxin or have later, neuroprotective actions. However, since oxidative stress, toxins, and pesticide exposure all have been shown to be involved in the pathogenesis of PD, GSTA4 and associated proteins are interesting candidates for both genetic analyses and as therapeutic targets. We chose to perform quantitative analyses on gene expression within the first week after lesion due to reports on increased *Gsta4* expression within days after injury [10], while cellular localization of GSTA4 was performed at time point chosen for histological analysis, 8 weeks post lesion. This leaves room for discussion on whether Gsta4 gene expression and GSTA4 protein localization are altered between early and late time points after the 6-OHDA lesion. In a future perspective involving clinical applications of these findings, the later, 8-week time point, which reflects a loss of dopamingeric neurons comparable to that of newly diagnosed to moderately affected PD patients, is of prime interest. Thus, future studies on the effects of astrocytic *Gsta4* expression on moderate to advanced stages of the neurodegenerative process could lead to new neuroprotective strategies.

In conclusion, our results identify *Gsta4* as an important factor regulating dopaminergic susceptibility to striatal 6-OHDA lesion. This makes GSTA4 highly relevant to study further in regards to the etiology and pathophysiology of PD.

## Supplementary Materials

The following are available online at www.mdpi.com/link/2076-3425/7/7/73/s1, Figure S1: *Gsta4* gene expression in the striatum and midbrain 7 days after striatal 6-OHDA lesion, Figure S2: Expression of GSTA4 in striatal astrocytes 8 weeks after 6-OHDA lesion.
