# Supplementary material for: Astrocytic Expression of GSTA4 Is Associated to Dopaminergic Neuroprotection in a Rat 6-OHDA Model of Parkinson’s Disease
Source: Brain Sci. 2017 Jun 26;7(7):73. doi: 10.3390/brainsci7070073 (PMC5532586; doi:10.3390/brainsci7070073)
Supplement: Supplementary file 1 [file brainsci-07-00073-s001.docx]

Article

Astrocytic expression of GSTA4 is associated to dopaminergic neuroprotection in a rat 6-OHDA model of Parkinson's disease

Michael Jewett^1^, Itzia Jimenez-Ferrer^1^ and Maria Swanberg^1,^*

^1^ Translational Neurogenetics Unit, Wallenberg Neuroscience Center, Department of Experimental Medical Science, Lund University, BMC A10, Sölvegatan 17, 221 84 Lund, Sweden

***** Correspondence: maria.swanberg@med.lu.se, Tel +46 (0)46 2220612

**Figure S1. *Gsta4* gene expression in the striatum and midbrain 7 days after striatal 6-OHDA lesion.** *Gsta4* expression is not significantly different in DA.VRA1 compared to DA for both the intact and the lesioned sides of the striatum (A) and midbrain (B). Gene expression levels are related to the mean value for DA at the ipsilateral (IL) side. Mean +/- SD are shown. One-way ANOVA. (DA= Dark Agouti; DA.VRA1= DA congenic; CL= Controlateral; IL=Ipsilateral)

** Figure S2**. **Expression of GSTA4 in striatal astrocytes 8 weeks after 6-OHDA lesion**. Immunofluorescent stainings of GSTA4 combined with cell-specific markers for (A) astrocytes; GFAP, (B) microglia; IBA1 and (C) neurons; NeuN. GSTA4 staining co-localized with GFAP (arrows in A) but not IBA1 (B) or NeuN (C), suggesting astrocytic expression. Pictures taken at 20x; scale bar=20μm. All markers were combined with nuclear marker DAPI (blue).
